# Supplementary material for: Dairy management practices associated with multi-drug resistant fecal commensals and Salmonella in cull cows: a machine learning approach
Source: PeerJ. 2021 Jul 16;9:e11732. doi: 10.7717/peerj.11732 (PMC8288115; doi:10.7717/peerj.11732)
Supplement: Supplemental Information 1 [file peerj-09-11732-s001.docx]

**Dairy management practices associated with multi-drug resistant fecal commensals and *Salmonella* in cull cows: a machine learning approach**

Pranav S. Pandit^1^, Deniece R. Williams^2^, Paul Rossitto^2^, John Adaska^3^, Richard Pereira^4^, Terry W. Lehenbauer^2,4^, Barbara A. Byrne ^5^, Xunde Li^6^, Robert Atwill^6^, Sharif S. Aly^2,4^*

^1^EpiCenter for Disease Dynamics, School of Veterinary Medicine, University of California Davis, Davis, CA, USA

^2^ Veterinary Medicine Teaching and Research Center, University of California, Davis, Tulare, CA, USA

^3^California Animal Health and Food Safety Laboratory System, University of California, Davis, Tulare, CA, USA

^4^Department of Population Health and Reproduction, School of Veterinary Medicine, University of California, Davis, Davis, CA, USA

^5^Department of Pathology, Microbiology & Immunology, School of Veterinary Medicine, University of California, Davis, California

^6^Western Institute for Food Safety and Security, University of California, Davis, Davis, CA, USA

*Corresponding Author: Sharif Aly; email address: [saly@ucdavis.edu](mailto:saly@ucdavis.edu)

**Supplementary Materials**

**
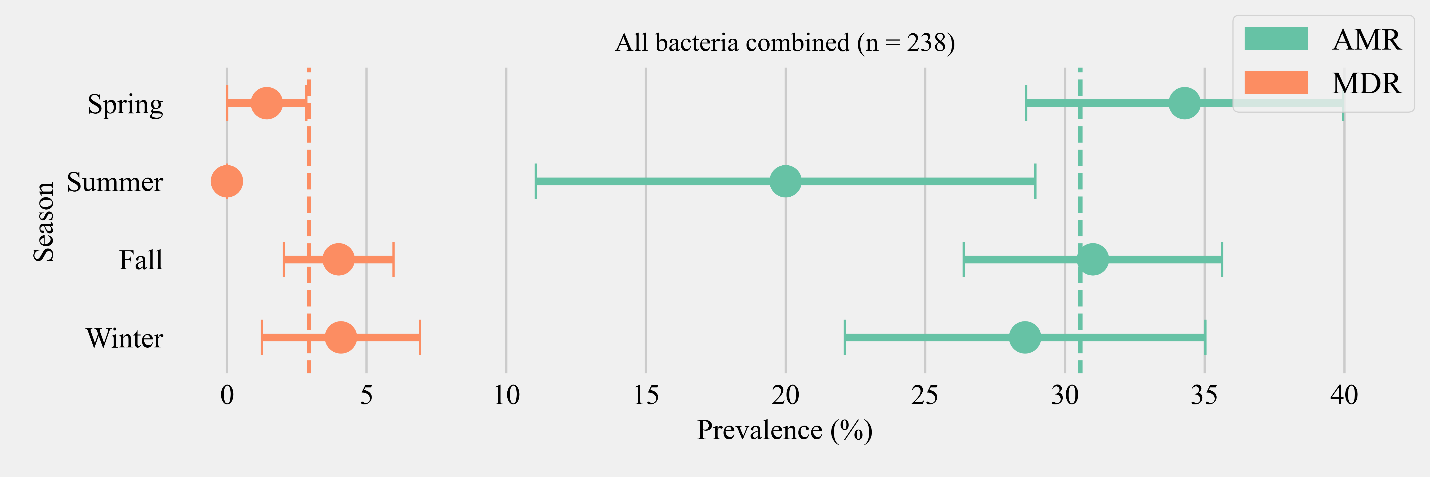
**

Figure S 1:: Seasonal variation in the prevalence of multidrug antimicrobial resistance (MDR), and antimicrobial resistance (AMR) phenotypes shed across Salmonella, E. coli, and Enterococcus spp. Orange and green dashed lines show the annual average prevalence of MDR and AMR in all six herds respectively. The proportion of cows that did not show any resistance are inverse of the sum of MDR and AMR proportions and not shown in the figure. Point estimates and single standard error deviation are represented by circles and whiskers respectively.


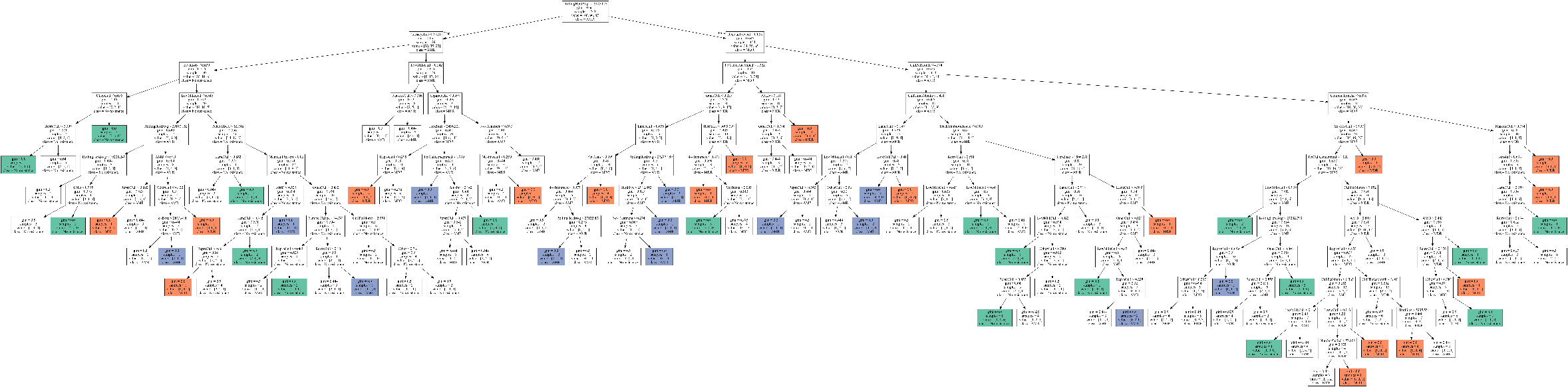


Figure S 2: Optimum decision tree to classify cows shedding multi-drug resistant (MDR), antimicrobial-resistant (AMR), and non-resistant Enterococcus sp., and E. coli (commensals) based on management practices observed in Californian dairy herds. Node boxes describe the decision point based on the management factor, followed by Gini impurity at the node, and the number of samples being classified at the node into three classes (value). Pure nodes are represented by colors. Green: Non-resistant, Blue: AMR, orange: MDR.


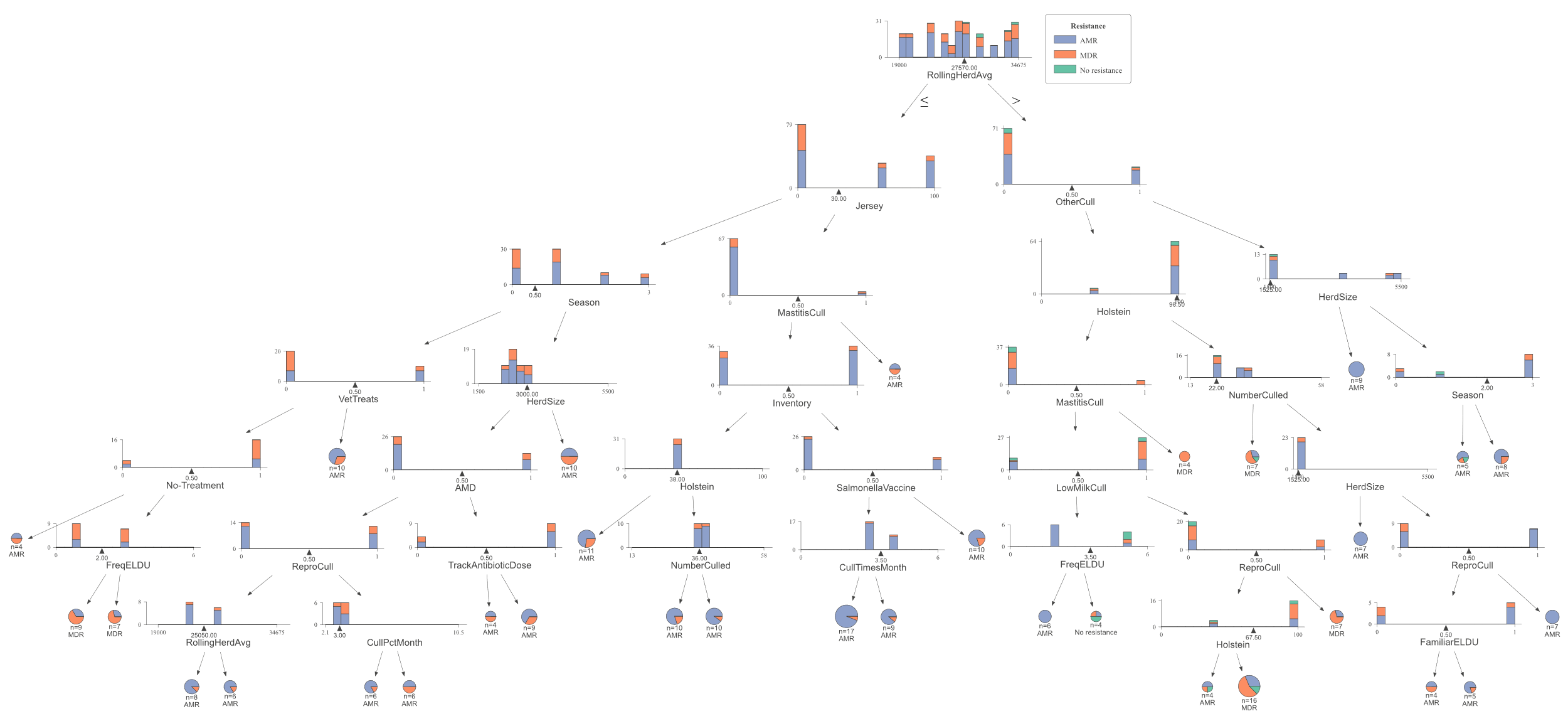


Figure S 3: Optimum decision tree to classify cows shedding multi-drug resistant (MDR), antimicrobial-resistant (AMR), and non-resistant E. coli based on management practices observed in Californian dairy herds. Node boxes describe the decision point based on the management factor, followed by Gini impurity at the node, and the number of samples being classified at the node into three classes (value). Pure nodes are represented by colors. Green: Non-resistant, Blue: AMR, orange: MDR.


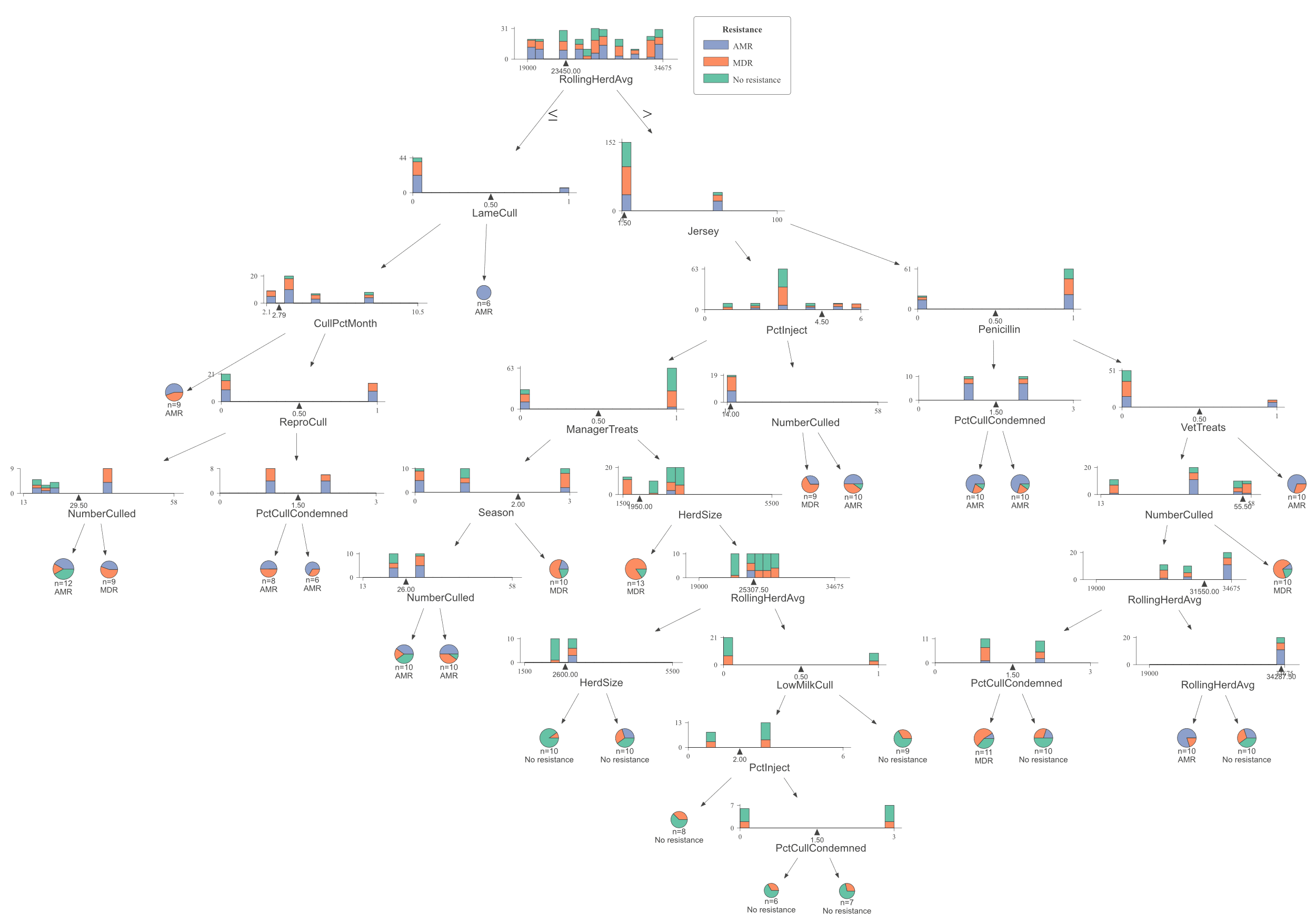


Figure S 4: Optimum decision tree to classify cows shedding multi-drug resistant (MDR), antimicrobial-resistant (AMR), and non-resistant, Enterococcus sp. based on management practices observed in Californian dairy herds. Node boxes describe the decision point based on the management factor, followed by Gini impurity at the node, and the number of samples being classified at the node into three classes (value). Pure nodes are represented by colors. Green: Non-resistant, Blue: AMR, orange: MDR.


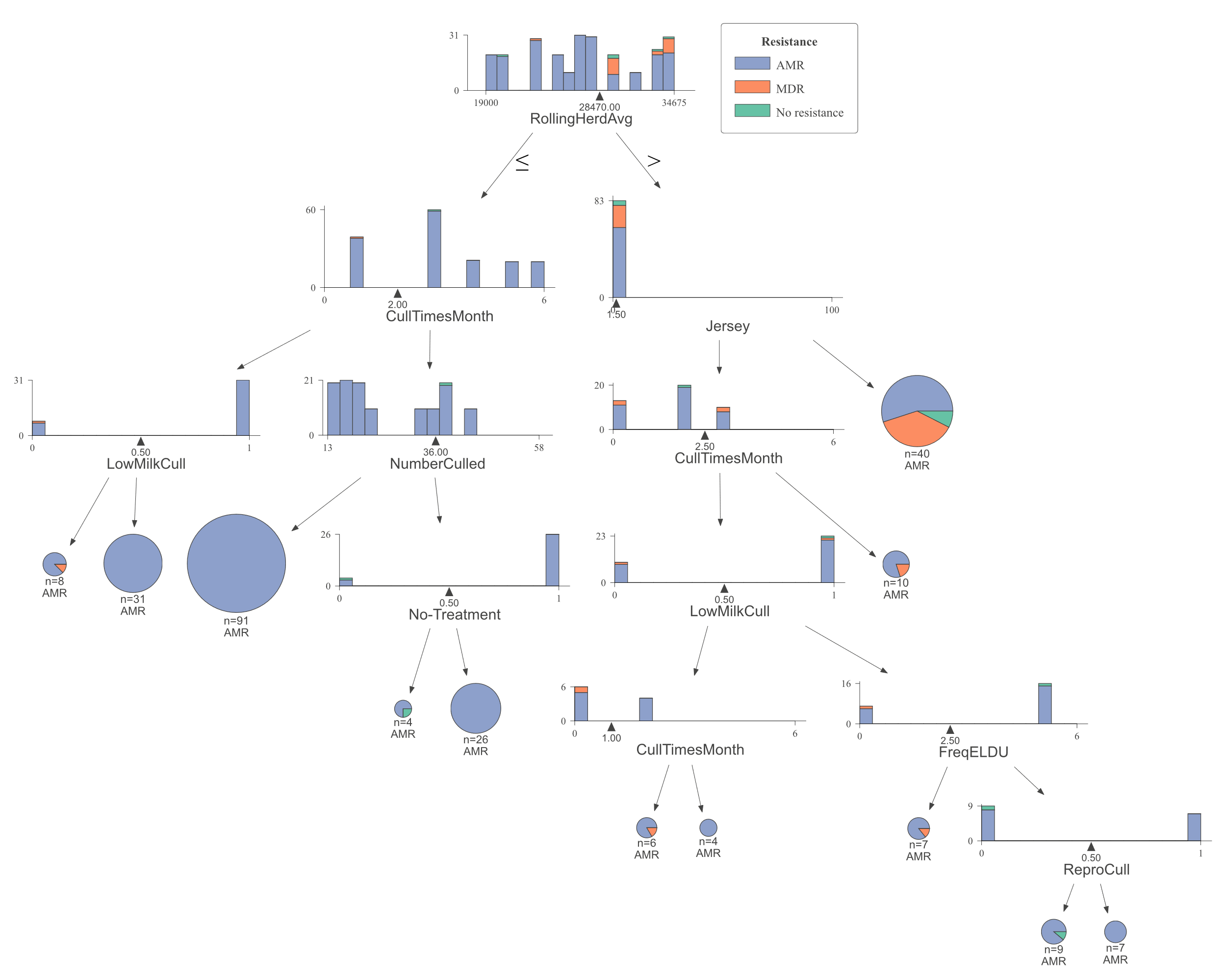


Figure S 5: Optimum decision tree to classify cows shedding multi-drug resistant (MDR), antimicrobial-resistant (AMR), and non-resistant Salmonella sp. based on management practices observed in Californian dairy herds. Node boxes describe the decision point based on the management factor, followed by Gini impurity at the node, and the number of samples being classified at the node into three classes (value). Pure nodes are represented by colors. Green: Non-resistant, Blue: AMR, orange: MDR.


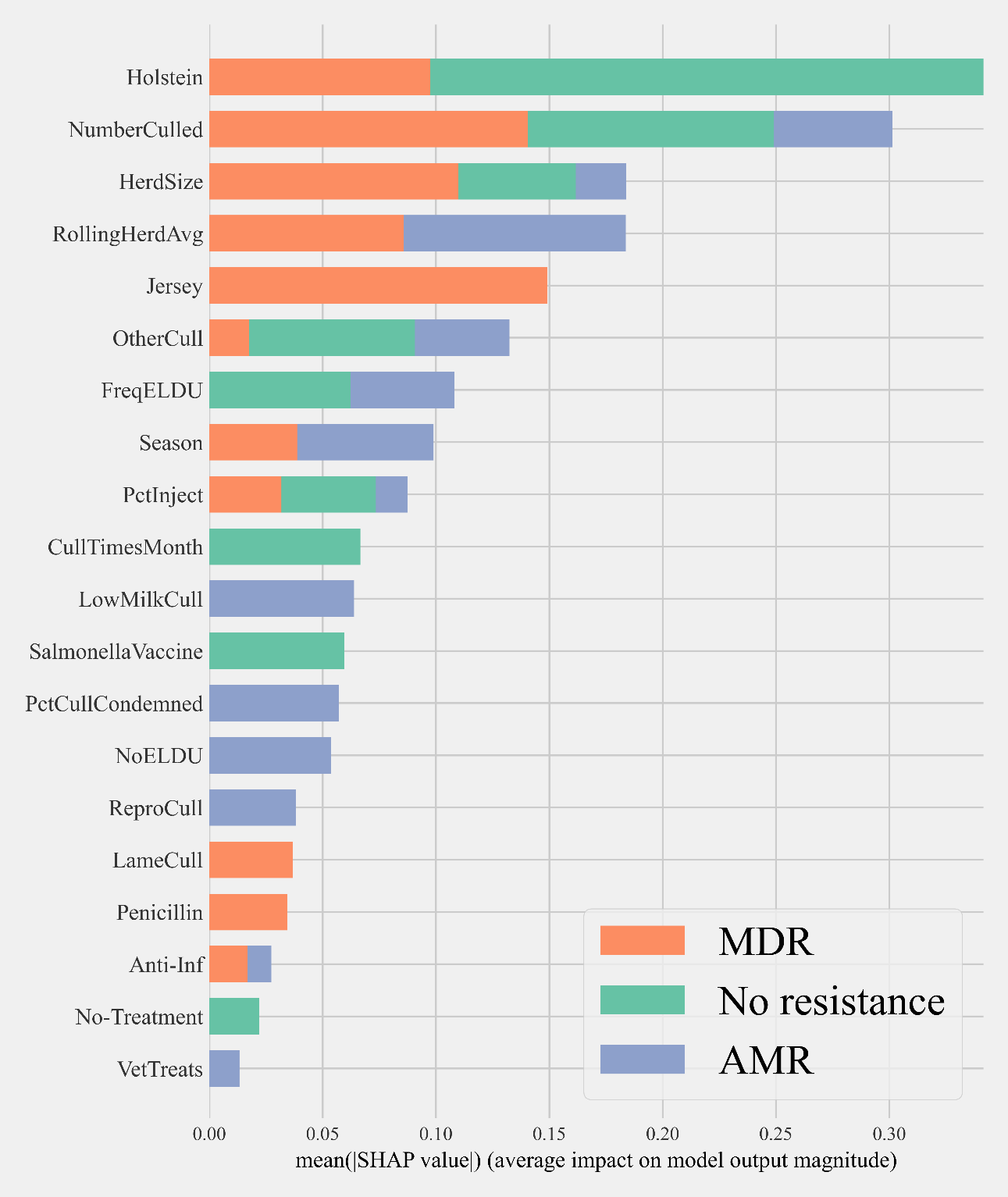


Figure S 6: Mean SHAP values depicting the impact of herd management practices on predicting multi-drug resistant Enterococcus sp. and E. coli (commensals) shed in dairy cows using Gradient boosting classification (XGboost) model (0: no resistance, 1: resistance for one or two antimicrobial classes, 2: multi-drug resistance)


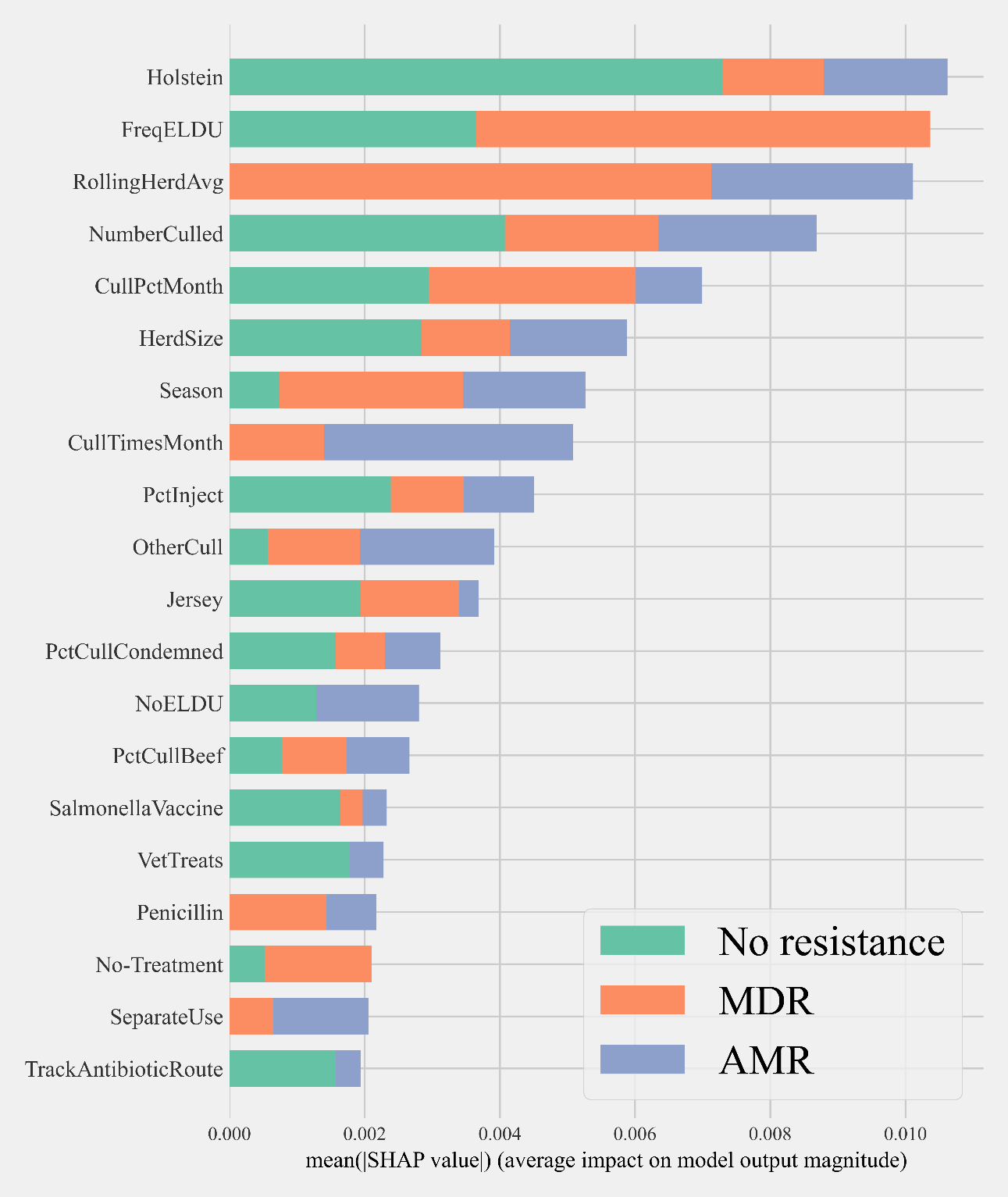


Figure S 7: Mean SHAP values depicting the impact of herd management practices on predicting multi-drug resistant Enterococcus sp. shed in dairy cows using Gradient boosting classification (XGboost) model (0: no resistance, 1: resistance for one or two antimicrobial classes, 2: multi-drug resistance)


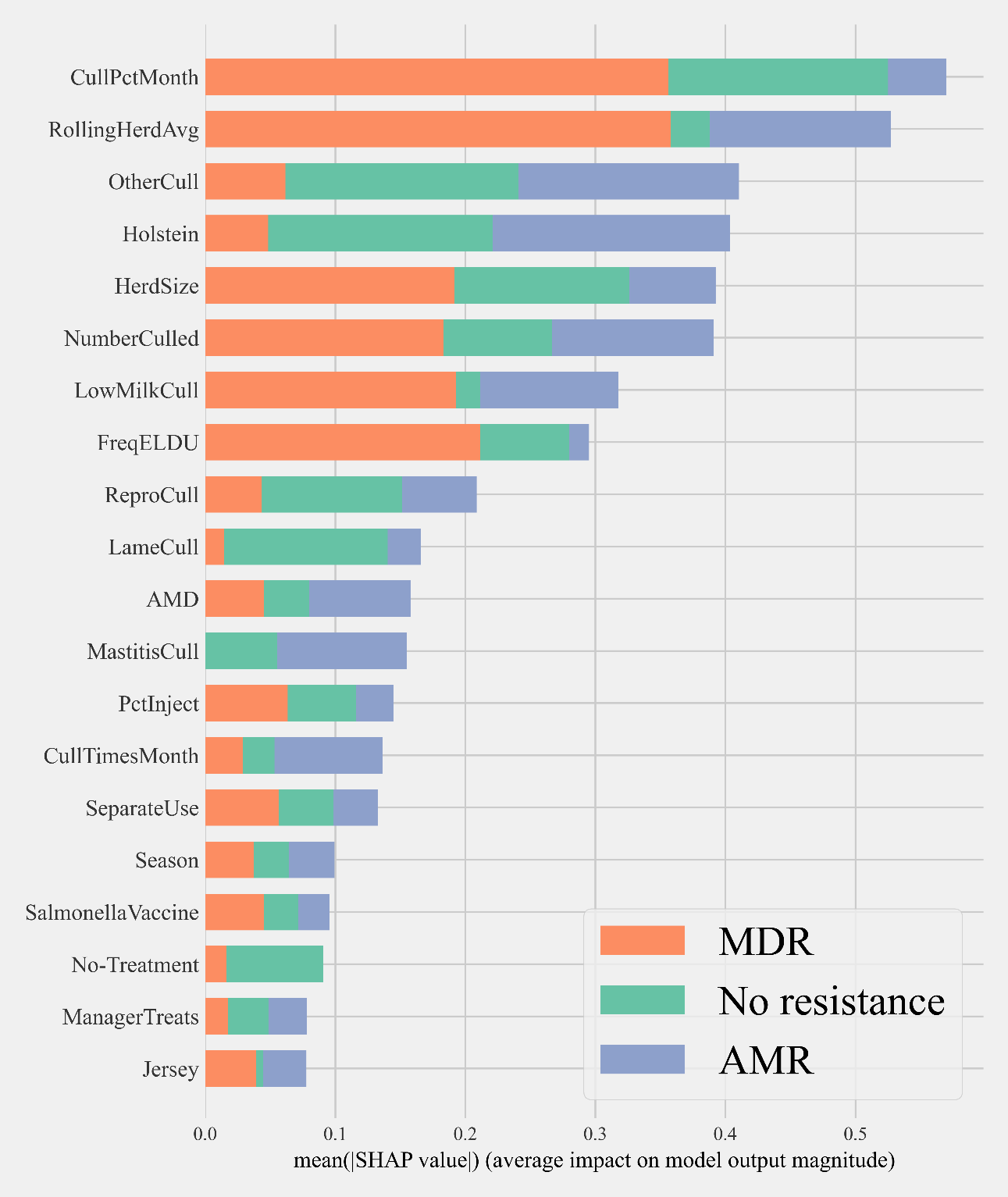


Figure S 8: Mean SHAP values depicting the impact of herd management practices on predicting multi-drug resistant E. coli shed in dairy cows using Gradient boosting classification (XGboost) model (0: no resistance, 1: resistance for one or two antimicrobial classes, 2: multi-drug resistance)
